# Monitoring of the Apple Fruit Moth: Detection of Genetic Variation and Structure Applying a Novel Multiplex Set of 19 STR Markers

**DOI:** 10.3390/molecules23040850

**Published:** 2018-04-08

**Authors:** Abdelhameed Elameen, Hans Geir Eiken, Ida Fløystad, Geir Knudsen, Snorre B. Hagen

**Affiliations:** 1 NIBIO, Norwegian Institute of Bioeconomy Research, Hoghskoleveien 7, N-1431 Aas, Norway; geir.knudsen@nibio.no; 2 NIBIO, Norwegian Institute of Bioeconomy Research, Svanhovd, N-9925 Svanvik, Norway; ida.floystad@nibio.no (I.F.); snorre.hagen@nibio.no (S.B.H.)

**Keywords:** *Argyresthia conjugella*, Lepidoptera, multiplex PCR, next-generation sequencing (NGS), tetranucleotide repeats

## Abstract

The apple fruit moth *Argyresthia conjugella* (Lepidoptera, Yponomeutidae) is a seed predator of rowan (*Sorbus aucuparia*) and is distributed in Europe and Asia. In Fennoscandia (Finland, Norway and Sweden), rowan fruit production is low every 2–4 years, and apple (*Malus domestica*) functions as an alternative host, resulting in economic loss in apple crops in inter-mast years. We have used Illumina MiSeq sequencing to identify a set of 19 novel tetra-nucleotide short tandem repeats (STRs) in *Argyresthia conjugella*. Such motifs are recommended for genetic monitoring, which may help to determine the eco-evolutionary processes acting on this pest insect. The 19 STRs were optimized and amplified into five multiplex PCR reactions. We tested individuals collected from Norway and Sweden (*n* = 64), and detected very high genetic variation (average 13.6 alleles, He = 0.75) compared to most other Lepidoptera species studied so far. Spatial genetic differentiation was low and gene flow was high in the test populations, although two non-spatial clusters could be detected. We conclude that this set of genetic markers may be a useful resource for population genetic monitoring of this economical important insect species.

## 1. Introduction

The apple fruit moth *Argyresthia conjugella* (Lepidoptera, Yponomeutidae; Zeller 1839) is a seed predator of rowan (*Sorbus aucuparia*). *Argyresthia conjugella* is small moth distributed in Europe and Asia [1,2], but the species has also been reported in North America [3]. The life cycle of *Argyresthia conjugella* is univoltine. This insect was detected as a crop pest for the first time in Norway in 1899 following an epidemic attack on apple [4]. Rowan also known as mountain-ash is a forest tree that grows up to 15 meters in height and belong to Rosaceae family. In Fennoscandia (Finland, Norway and Sweden), rowan fruit production shows large fluctuations and is low every 2–4 years [5]. In these inter-mast years, female apple fruit moths are forced to search for alternative hosts and nearby apple (*Malus domestica*) plantations are especially vulnerable [5,6,7]. Rowan and apple are both rosaceous plant and their fruits give off many of the same volatile compounds. These similarities may be the reason for the *Argyresthia conjugella* host switch from rowan to apple [6]. For this reason, the apple fruit moth is regarded as a serious insect pest of apple crops in Fennoscandia and can in extreme years devastate the entire apple crop [5]. In Norway, reduced pesticide use against *Argyresthia conjugella* was achieved by introducing a forecasting system that monitors rowan masting, the moth population, and its natural enemies [8]. Despite its economic importance, the genetic structure, diversity, genetic exchange and the eco-evolutionary processes, which act on this insect pest remain to be investigated [9].

Genetic diversity is influenced by several factors, such as gene flow, population fragmentation, inbreeding and geographical isolation. Adult butterflies and moths may have long distance migration [10,11], but this is not always the case [12,13,14]. Genetic drift alone may act on insect populations, resulting in genetically distinct populations, due to reproductive isolation, or interruption of gene flow among regions [15]. Further, reproductive isolation is impacted by the population size, individual dispersal, geographic isolation, and local ecological adaptation [16,17,18,19]. Dispersal is a key factor in both population dynamics and genetic exchange [20]. Continuous dispersal helps long-term survival of a species and can lower the genetic drift rate of local populations [21]. Gene flow and dispersal may be studied using genetic markers, such as short tandem repeats (STRs). STRs are highly polymorphic and reproducible co-dominant markers [22,23] and the efficiency and accuracy are superior to other dominant markers [9]. Short repeat motifs, long major allele, unbroken repeat, and localization in non-coding regions are associated with high STRs variability [24]. However, tri- and tetranucleotides are preferred for genetic monitoring due to higher reproducibility compared to dinucleotide STRs [25].

The development of STRs for Lepidoptera was known to be labor-intensive, expensive, and difficult, due to the proximity between STR sequences, repetitive sequences in the flanking regions and high frequencies of null alleles [26,27]. However, in the last few years, next-generation sequencing has provided a cheaper, faster, and efficient method for developing STR markers [28,29,30].

In our previous study [9], we used AFLP markers to investigate the genetic diversity of *A. conjugella*. Consequently, this study aimed to (i) identify tetranucleotide polymorphic STR markers using Illumina MiSeq sequencing; (ii) develop multiplex PCR combinations of these STR markers; (iii) test the cross-amplifications of the markers on related species (*Argyresthia ivella*, *Argyresthia pruniella* and *Argyresthia curvella*) and (iv) evaluate the statistical strength of the markers in a test population. The overall aim of this methodological study is to develop a quality set of genetic markers that can be used to study the genetic diversity and to understand the evolutionary process acting on *Argyresthia conjugella*. In turn, such knowledge may influence the management of this pest that has large impact on yield apple production.

## 2. Results

Illumina MiSeq sequencing of five *Argyresthia conjugella* individuals from various locations in Norway and Sweden gave a total of 21,638 contigs and singlets, of which 2918 showed variable repeats. We were able to develop suitable primers in 2374 STR fragments. Based on the sequences from the same five individuals, we selected the 40 tetranucleotides showing the highest variability. Next, we tested these 40 tetranucleotide markers on 15 individuals (not shown). From this result, we selected the 19 markers with highest PCR efficiency and genetic variation for further evaluation. The results obtained from PCR sequencing identified the STR repeat motifs (Supplementary Table S3 and Table 1). A population of 64 different *Argyresthia conjugella* individuals (Supplementary Table S1) were used for further analysis.

**Figure 1:**
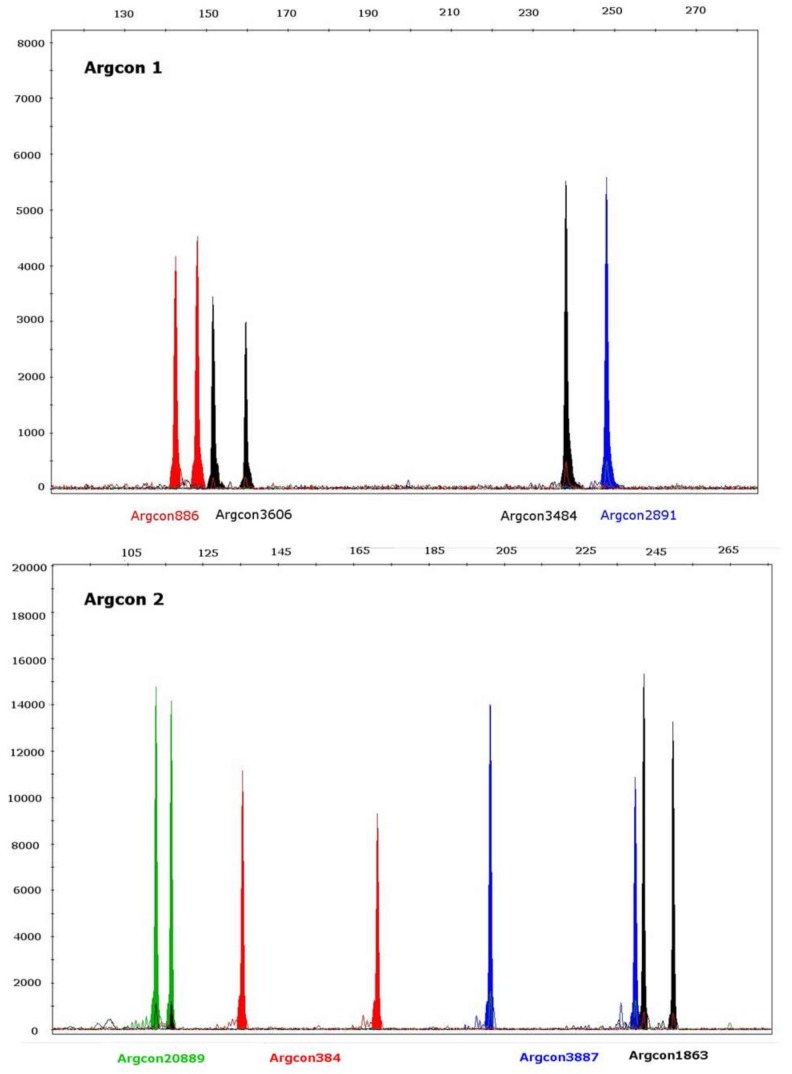

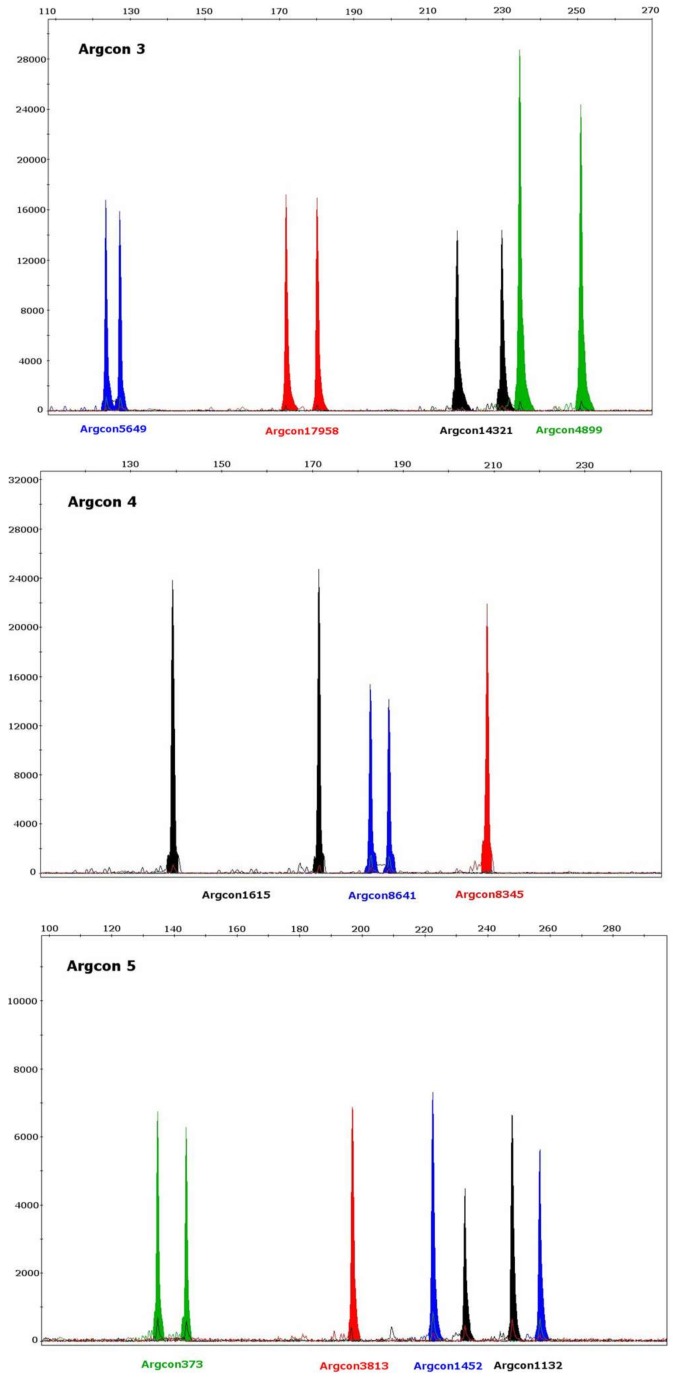
Chromatograms from capillary electrophoresis showing panels of the five multiplex PCRs (Argon 1 to Argon 5) for the 19 tetranucleotide STRs from the apple fruit moth (*Argyresthia conjugella*).

All 19 singleplex PCRs of the selected tetranucleotide markers were successfully amplified (singleplex PCRs were repeated two times with seven of the 64 *Aryresthia conjugella* individuals and confirmed the same amplification results). The 19 STR markers were then organized into five PCR multiplex panels, to make the genotyping faster and more cost-effective. The STR markers were designed and organized to adjust the PCR product size for the multiplex-PCR. PCR product for each locus was labeled with one of the four different fluorescent labels (6FAM, NED, PET and VIC) so that no two markers with the same fluorescent dye had overlapping allele size ranges. The multiplex PCR combinations were optimized (not shown), and examples of the chromatograms from the capillary electrophoresis runs are shown in Figure 1. An overview of the combinations of PCR primers, primer concentrations and fluorescent labels for the five resulting multiplex panels is given in Table 2. The multiplex panels showed that the 19 loci produced one major peak for each allele, except for one marker (Argcon-1452) where only 38 of 64 individuals could be genotyped due to difficult allele designations (Table 3).

The observed and expected heterozygosity obtained in the study varied from 0.1455 to 0.9074 and 0.4816 to 0.9399, respectively. The mean expected heterozygosity was He = 0.75, Nei’s genetic diversity index ranged from 0.4763 to 0.9399, and estimated F_IS_ was 0.0486 to 0.6952 (Table 3).

We also tested the 19 STRs using DNA from three species from the same genus; *Argyresthia ivella*, *Argyresthia pruniella*, and *Argyresthia curvella*. Cross-amplification could only be detected for two of the 19 STRs (*Argyresthia ivella*: Arg1452 and, *Argyresthia pruniella*: Arg4899).

**Figure 2:**
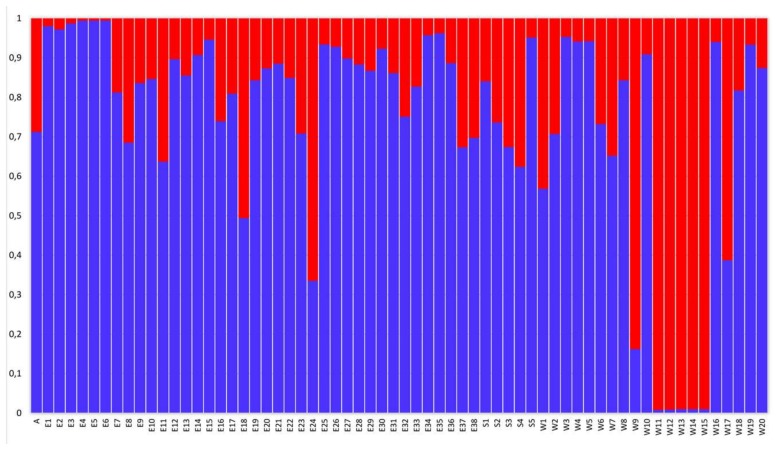
Population genetic structure analyses of 64 *Argyresthia conjugella* individuals using STRUCTURE. The insects are sorted according to Id number as in Supplementary Table S1 (W: Norway: Western region, E: Norway: Eastern region and S: Sweden).

Bayesian clustering analysis with STRUCTURE software (see Materials and Methods) assigned the 64 *Argyresthia conjugella* individuals in two potential clusters that were not geographically restricted to specific regions (Figure 2 and Supplementary Table S1).

The STRs data were also analyzed using the analysis of molecular variance (AMOVA) and variance components were estimated (Table 4). As shown in Table 4, higher genetic variation was found within (97.76%) than among the geographic regions (2.24%). Low F_ST_ values (Table 5) were estimated among the three sampling locations (average 0.0224), while a substantially higher F_ST_ value (0.4034) was found between the two clusters indicated in Figure 2. In general, and when not considering the genetic clustering, estimated gene flow among the regions was found to be high (Nm = 10.91).

## 3. Discussion

We have developed 19 novel tetranucleotide STR markers to be used in multiplex PCRs for population genetic monitoring of the apple fruit moth, which is a threat to apple crops in Fennoscandia. To our knowledge, this is the first full set of tetranucleotide STR markers that have been developed for an insect species. Tetranucleotide STRs are usually less variable than dinucleotides [24], but are recommended for genetic monitoring due to higher reproducibility and performance [25]. When applying the 19 novel tetranucleotide STRs to a test population of 64 individuals, we found high genetic variation compared to other Lepidoptera species studied so far. In addition, substantial gene flow among geographical regions was detected in our limited test population. Non-spatial genetic structure could also be detected, and the two genetic clusters were not restricted to geographical areas.

Single PCR of many fluorescent-labeled STR markers is time consuming, costly and represents a risk of cross species amplifications. To overcome this, multiplex PCR provides a high throughput technique that will reduce the cost and increase species specificity [32,33]. Few other studies have applied STRs multiplex-PCRs to Lepidoptera species, although notable exceptions exist; 17 STRs from *Thaumetopoea pityocampa*, with three multiplex-PCRs [34]; and 21 STR markers from *Epirrita autumnata*, with six multiplex-PCRs [35]. The five multiplex PCRs in our study were successfully amplified and the 19 STRs were highly polymorphic. However, the STR marker Argcon-1452 showed a low success rate and may be excluded from large studies. Cross-amplifications to related species were detected in single cases for two different markers. However, the multiplex approach ensured the species specificity for our method.

In this study, we observed a very high number of alleles per locus (average = 13.6) compared to other Lepidoptera species; 2.2 in *Helicoverpa zea* [36], 3 in *Hyphantria cunea* [37], 4.1 in *Helicoverpa armigera* [36], 4.6 in *Carposina sasakii* [38], 4.7 in *Epirrita autumnata* [35], 4.7 in *Plutella xylostella* [39], 7.0 in *Chilo suppressalis* [40], 8.8 in *Zeiraphera diniana* [41], and 10.5 in *Parnassius apollo* [42]. The large number of STR markers detected (2374) in this study enabled the possibility of selection of the most highly polymorphic markers. In addition, our finding of high allelic variation may also be due to high genetic diversity among *Argyresthia conjugella* populations which have not been investigated before using STRs.

STRs analyses of *Argyresthia conjugella* showed very high expected heterozygosity (He = 0.75): higher than previously reported using AFLPs markers (He = 0.31 [9]). However, AFLPs and STR markers are not directly comparable. The expected heterozygosity of *Argyresthia conjugella* in our study, was slightly higher than in SSR analyses of *Metrioptera roeselii* ‘0.61’ [21], *Leptomyrmex pallens* ‘0.51’ [43] and *Cerambyx cerdo* ‘0.63’ [44]. However, the expected heterozygosity was much higher than was previously detected by SSRs in *Plutella xylostella* ‘0.35’ [11], *Anopheles nuneztovari* (Culicidae) ‘0.34’ [45], *Glossina pallidipes* ‘0.35’ [46], *Bombus distinguendus* ‘0.38’ [47] and *Epirrita autumnata* ‘0.43’ [35]. We may speculate if the high genetic variation may be caused by previous population fragmentation and/or multiple founders as a result of the life cycle, outbreak cycles, lower migration distance as well as the topography in Scandinavia and/or the use of insecticides on selected parts of the population.

Our genetic analyses showed two potential main clusters, but without any structure among the geographical regions. Two clusters were also found in our previous study of the apple fruit moth in Norway [9], but the clusters were associated with geographical locations separated by a mountain plateau. Genetic differentiation (F_ST_) was found to be low in this study, indicating high gene flow among the geographical areas, which has also been reported in other Lepidoptera species (e.g., *Plutella xylostella*, *Cydia pomonelle* and *Rhagoletis indifferens*) [48,49,50,51]. In contrast, other studies (for *Argyresthia conjugella*, *Boloria eunomia*, *Sesamia nonagriodes Diatraea saccharalis* and *Cydia pomonelle*), have documented substantial genetic differentiation and low gene flow rate among geographical regions [9,52,53,54,55]. In our study, the samples from Sweden clustered together with the samples from Norway, which further supports the existence of substantial gene flow for the apple fruit moth in Scandinavia. Conclusively, both the genetic differentiation and the structural analyses supported the existence of high gene flow among the different regions.

Previously, genetic diversity of insect species has demonstrated the existence of genetic differentiation between insecticide susceptible and resistant populations [49,56]. The two clusters found in this study and as well in our previous study [9], may also be due to different management regimes.

The high gene flow detected in the study, may be helpful both in determining sources from which *Argyresthia conjugella* disperse and for monitoring insecticide resistance and may be a key factor for successful and stable control of insect pests.

In conclusion, we have developed 19 highly polymorphic tetranucleotide STR markers for *Argyresthia conjugella*, and the multiplex PCR sets for these STR markers will enable fast and wide population genetic studies of the species. These novel STRs may be used on larger scales to investigate the genetic diversity and population structure of this economical important insect species. Understanding the evolutionary process acting on *Argyresthia conjugella* may provide sufficient and efficient methods for management of this pest, which has large impact on yield in apple production.

## 4. Materials and Methods

### 4.1. Sampling

**Figure 3:**
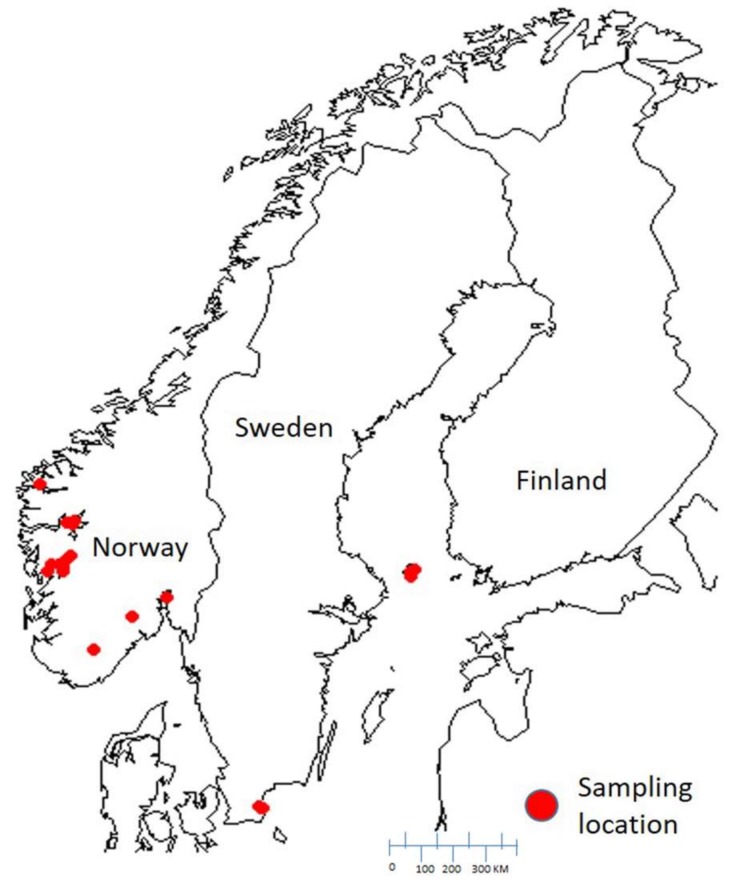
The geographical locations of the sampling areas. The sampling of *Argyresthia conjugella* was based on their exact global position coordinates (GPS).

In autumn 2015, *Argyresthia conjugella* larvae were collected from infested rowan berries. The berries were collected from multiple trees per field from 15 different locations. *Argyresthia conjugella* larvae were stored over rolls of corrugated cardboard, where larvae went into pupal diapause. A total of 64 individuals (females and males) were sampled from Eastern and Western regions in Norway, and one small population from Sweden (Figure 3). The samples were stored at −80 °C until DNA was extracted. The list of samples used in this study are provided in supplementary Table S1.

### 4.2. Identification of STRs

The extraction of DNA from *Argyresthia conjugella* samples was performed using DNeasy Blood and Tissue Kits (Qiagen, Tokyo, Japan) according to the manufacturer’s instructions.

Illumina MiSeq sequencing was used to identify STR markers. Briefly, the construction of the MiSeq libraries was done by using 5 individuals of *Argyresthia conjugella* that were equimolar and pooled together. Five μg genomic DNA was digested using ultrasonic bath end-repaired and A-tailed. DNA fragments were then ligated using Illumina adapters i501/i701. DNA fragment enrichment for STR availability was done using magnetic streptavidin beads and biotin-labeled GATA and GTAT repeat oligonucleotides. The enrichment STR library was analyzed using Illumina MiSeq sequencing using the 2 × 250 v2 format. The paired end reads were merged with FLASH 1.2.9 software [57]. Then, a de-novo assembly was performed with the merged paired end reads with MIRA 4.0.1 software [58]. This software classifies assembled sequences into contigs and singlets. Reads that have no overlap with any other reads become singlets (only 2 singlets were detected and do not contained a microsatellite insert). A total of 21,638 contigs were screened. Detection of polymorphic STR alleles was done by assembling the different contigs using Tandem Repeats Finder, v 4.09 software [59]. The library analyses showed 2918 contigs with variable microsatellites repeats that contained a minimum of six repeat units of tetranucleotide and trinucleotide and ten repeats of dinucleotide. We were able to develop suitable primers in 2374 fragments using primer 3 version 4.0 [60]. Among these sequences, 40 tetranucletide loci were selected to test the primer amplification efficiency across 15 *Argyresthia conjugella* individuals from various locations in Norway and Sweden. Of these tested markers, 19 highly polymorphic STRs were selected for further experiments.

### 4.3. PCR Amplification

PCR primers for the 19 loci were optimized using OligoPerfect™ Designer (Applied Biosystem, Foster City, CA, USA), with the following criteria: (i) length of PCR product should be as short as possible and between 90 and 300 bp; (ii) flanking regions should not contain a mononucleotide stretch of more than five bases; (iii) annealing temperature were optimized to fall between 56 °C and 63 °C; and (iv) difference in temperature between forward and reverse primer should not exceed 2 °C. The forward-primers were labeled with one of four fluorescent dyes (6FAM, VIC, NED or PET). The dye colors were assigned to the primers in a fashion that allowed for different dyes for the markers with assumed overlapping allele ranges. In addition, we added PIG-tailing sequences (GTTTCTT) to the reverse primers [31] in the 5′ end of the primer to facilitate accurate genotyping.

Single PCRs were initially performed on seven individuals for each of the 20 primer pairs, in a 10 µL reaction volume containing 1× PCR Gold buffer (ABI), 200 µM dNTP (Eurogentec, Liege, Belgium), 1.5 mM MgCl2 (ABI), 0.5 µM of each primer (Life technologies, Carlsbad, CA, USA), 1 U Amplitaq Gold DNA polymerase (ABI), 1× BSA (NEB) and 1 µL template (0.1 to 2.0 ng). PCR reactions were performed on a 2720 Thermal cycler and the conditions for the PCR was 10 min at 95 °C, 26 cycles of 30 s at 94 °C, 30 s at 58 °C, 1 min 72 °C and final extension for 45 min at 72 °C.

PCR products (1 µL) were then mixed with Genescan 500 LIZ (Applied Biosystems) size standard (0.24 µL) and Hi-Di formamide (10.00 µL), following a 2 min denaturation at 95 °C on a 2720 Thermal cycler. Capillary electrophoresis was carried out on an ABI 3730 DNA Analyzer (Applied Biosystems). The POP-7™ Polymer was used as a separation matrix and the sample injection time were set to 4 s/2 kv. PCR fragments were analyzed in GeneMapper 4.1 (Applied Biosystems). The singleplex PCR reactions were carried out to verify the successful amplifications of the markers. One of the STR-markers (Arg4244) did not successfully amplify and were left out of further multiplex-PCR development.

For multiplex development, different combinations of the STR-markers were tested until a combination of four markers amplified successfully with clear chromatograms and without artifact alleles/spikes/primer dimer. PCR product for each locus was labeled with one of the four different fluorescent labels (6FAM, NED, PET and VIC) in such a manner that no two markers with same fluorescent dye had overlapping allele size ranges. The concentration of the primers were adjusted so that the markers within a multiplex panel were equal in height (RFU), and the number of cycles for the PCR-reaction were adjusted to achieve optimal peak height of the alleles (between 8000–24,000 RFU).

For the final analysis, the 19 STRs were split into four tetraplex panels (multiplex I, II, III and V) and one triplex panel (IV). The PCR reactions were carried out in 10 µL reaction volume: 5 µL 2× multiplex PCR master mix (Qiagen Multiplex kit), 0.05 µg/µL BSA (NEB) and adjusted primer set concentrations (Table 2). PCR conditions for multiplex I, II and IV were 10 min at 95 °C, 24 cycles of 30 s at 94 °C, 30 s at 58 °C, 1 min 72 °C and final extension for 45 min at 72 °C. PCR conditions for multiplex III and V were 10 min at 95 °C, 25 cycles of 30 s at 94 °C, 30 s at 60 °C, 1 min 72 °C and final extension for 45 min at 72 °C. The fragment analyzes for the multiplex reactions were carried out as described by the singleplex reactions.

To check for possible contamination, negative controls were included for every seventh sample in all measurements in this study. Negative controls for both single and multiplex PCRs were carried out with all of the PCR master-mix components except the DNA template (water was added instead of DNA). The seven samples of *Argyresthia conjugella* used for the initial testing and optimization of the single- and multiplex runs were used as positive controls.

### 4.4. Species Specificity

A test for cross-species amplification was carried out by testing the STRs on other closely related species of *Argyresthia*: *A. ivella*, *A. pruniella* and *A. curvella*. All the nineteen markers were tested against two DNA samples for each of the abovementioned species. The PCR and STRs analyses were performed as described for *Argyresthia conjugella*. The STR-markers were considered species specific if they did not amplify in any of the related species.

### 4.5. Data Analyses

We calculated the deviations from Hardy–Weinberg equilibrium (HWE) for each single locus of these polymorphic STRs studied. The number of alleles, allele frequencies, expected-observed heterozygosity and inbreeding coefficient (F_IS_) were calculated. These analyses were performed using Genepop 4.0 software [61]. We calculated Nei’s genetic diversity [62] using Popgene version 1.32 [63].

Bayesian clustering approach of genetic mixture analysis (Structure 2.3.4 software) was carried as developed by [64] to identify the number of genetically homogeneous clusters (K) (Figure S1). Plots of likelihoods, similarity coefficients and ΔKs [64] were made with Structure Harvester [65], as published by [9].

We performed an analysis of molecular variance (AMOVA) [66] to study the genetic diversity between clusters and among the different regions. Genetic differentiation and F_ST_ values were estimated using Arlequin software, version 2.000 [67], as published by [9]. Gene flow was calculated as published by [68].

## Figures and Tables

**Table 1:** Overview of the 19 tetranucleotide short tandem repeats (STRs) identified in the apple fruit moth (*Argyresthia conjugella*). Asterisk = number of repeats from sequencing.

Locus	Size Range (bp) Illumina MiSeq *n* = 5	Size Range (bp) ABI3730 Capillary Electrophoresis *n* = 64	Repeat Motif (Illumina MiSeq) *
Argcon373	97–153	98–246	(TTGT)_22_
Argcon384	121–196	129–207	(AACA)_10_
Argcon886	119–137	129–201	(TTTC)_7_
Argcon1132	205–247	210–268	(AGAA)_7_
Argcon1452	214–247	208–292	(TCTT)_9_
Argcon1615	120–164	129–169	(TTTG)_10_
Argcon1863	231–246	235–249	(ACAT)_7_
Argcon2891	239–251	237–258	(TATC)_7_
Argcon3484	230–251	230–258	(TTGT)_8_
Argcon3606	143–160	151–183	(ACAT)_9_
Argcon3813	179–199	188–216	(TTCT)_7_
Argcon3887	185–261	188–340	(AAGA)_9_
Argcon4899	217–273	214–386	(TTCT)_9_
Argcon5649	105–140	111–151	(AAAC)_7_
Argcon8345	196–256	204–260	(TTTC)_8_
Argcon8461	120–242	122–306	(TTTC)_16_
Argcon14321	199–228	205–233	(TATC)_8_
Argcon17958	154–192	164–284	(GTTT)_9_
Argcon20889	97–143	104–156	(TTTC)_12_

**Table 2:** Five multiplex PCR panels (I to V) for the 19 STRs from *Argyresthia conjugella.* Primer combinations, primer concentrations and fluorescent dye for the STRs used in each multiplex reaction.

Multiplex Panel	Locus	Primer Sequences (5′-3′) ^a^	PCR Concentration, Dye	Gene-Bank Accession No.
**I**	Argcon_3606	F: AGTGAACCTACTGAGCGTCC	0.05 µM, NED	MF156559
		R: GTTTCTTCCCTTATGGGAAAAGGCGTG		
	Argcon_3484	F: GGGCAGCTGTTTCCCAATTC	0.10 µM, NED	MF156558
		R: GTTTCTTCTCCTCGTGCATTTTTGGG		
	Argcon_2891	F: AGAACTGGGCCTCACGATAC	0.10 µM, FAM	MF156557
		R: GTTTCTTGTTATCGGCATTCCACAAGGG		
	Argcon_886	F: ACCCGACCTGAACATATCCG	0.10 µM, PET	MF156552
		R: GTTTCTTCCATCGTTGGCACTTACGAG		
**II**	Argcon_20889	F: TGTGTCTAGTTTCTTGTATTTGTTGC	0.10 µM, VIC	MF156568
		R: GTTTCTTTAGTGTGGGCTAAGGGATGC		
	Argcon_384	F: CATGTCTCCTCTTTGCAGCG	0.15 µM, PET	MF156551
		R: GTTTCTTGTAAGGGAGTGTCGTGTTGC		
	Argcon_1863	F: CGCCCCGGATTCTCAACTAC	0.20 µM, NED	MF156556
		R: GTTTCTTTCACCCCTCTCTGTATTCGTC		
	Argcon_3887	F: CATTGTTGACAGCTCGGCAC	0.25 µM, FAM	MF156561
		R: GTTTCTTGTGAGCCTTTCGGATTTGGG		
**III**	Argcon_14321	F: TGTTTTGTTCAATCTGTATTACTTGTC	0.15 µM, NED	MF156566
		R: GTTTCTTACAGGGGGACAATCCAATCTAC		
	Argcon_5649	F: AGCCCTACGACTCCATCAAC	0.10 µM, FAM	MF156563
		R: GTTTCTTGCTAAACTATCCGTCGGCAC		
	Argcon_17958	F: GCTCAGTGTATCAGGTACGAG	0.20 µM, PET	MF156567
		R: GTTTCTTCGCTGTTCTACATGGAGCTG		
	Argcon_4899	F: TCATGGTTTGGCATGTCGAG	0.10 µM, VIC	MF156562
		R: GTTTCTTAGTTCAAATCCGTCCTAAAAGC		
**IV**	Argcon_1615	F: CCCCTTATTTGAGCAGTTGAGC	0.05 µM, NED	MF156555
		R: GTTTCTTGCAACATTATTTCGTCCGCAG		
	Argcon_8345	F: GCTCAAACGGTTGTCCCTAC	0.20 µM, PET	MF156564
		R: GTTTCTTGTATGCTACGGTTACAGGGC		
	Argcon_8461	F: ACTTTACTGGCCTAGGTGCG	0.15 µM, FAM	MF156565
		R: GTTTCTTCCAGGTGAAACATCGTGAGG		
**V**	Argcon_373	F: AGTACCTCGTCGATACGCAC	0.05 µM, VIC	MF156550
		R: GTTTCTTAGGGGTGTCAGGATGTGATG		
	Argcon_3813	F: GCTGTCGTAAACCCTTCCAC	0.10 µM, PET	MF156560
		R: GTTTCTTGGCCCCATTGGTTCCATAAC		
	Argcon_1452	F: AATAACAGTGGCACACCACG	0.15 µM, FAM	MF156554
		R: GTTTCTTTATGCGGATTCCAAACGCAG		
	Argcon_1132	F: TGTCTATGGAAGCCCCGATG	0.15 µM, NED	MF156553
		R: GTTTCTTGTTCCAAGATTTGCCGCTCC		

^a^ F forward, R reverse (5′ end GTTTCTT-tail on all R-primers [31]).

**Table 3:** Basic statistics of 19 STRs loci developed for *Argyresthia conjugella* in a survey of 64 individuals from Norway and Sweden.

Locus	N_A_	N_G_	H_O_	H_E_	Nei	F_IS_	HWE *p* Values ^a^
Argcon2891	10	63	0.3333	0.6060	0.6012	0.4455	0.0087 **
Argcon3606	7	64	0.6000	0.6136	0.6089	0.0146	0.9709
Arg3484	11	64	0.3750	0.7826	0.7765	0.5171	0.0016 **
Arg886	6	64	0.5156	0.4801	0.4763	−0.0825	0.0093 **
Arg3887	26	59	0.4464	0.9484	0.9399	0.5250	0.0007 **
Arg20889	8	64	0.6094	0.6380	0.6331	0.0374	0.0004 **
Arg1863	7	55	0.1455	0.4816	0.4772	0.6952	0.0001 **
Arg384	16	62	0.7097	0.7578	0.7517	0.0559	0.0002 **
Arg5649	11	64	0.4531	0.8108	0.8044	0.4367	0.0753 *
Argcon4899	20	59	0.5714	0.8465	0.8390	0.3189	0.0001 **
Argcon14321	8	64	0.6094	0.7464	0.7406	0.1772	0.1467
Argcon17958	17	64	0.7231	0.7659	0.7600	0.0486	0.0035 **
Argcon8461	24	58	0.9074	0.9084	0.9000	−0.0082	0.2887
Argcon1615	14	57	0.5789	0.8871	0.8793	0.3416	0.0076 **
Argcon8345	18	62	0.5484	0.8581	0.8512	0.3557	0.0012 **
Argcon1452	16	38	0.5833	0.9221	0.9093	0.3585	0.0127 *
Argcon373	21	62	0.8548	0.9340	0.9265	0.0774	0.0016 **
Argcon1132	11	62	0.6129	0.6808	0.6753	0.0924	0.0187 *
Argcon3813	8	63	0.3387	0.5507	0.5463	0.3800	0.9939
Mean	13.6	60.2	0.5535	0.7484	0.7419	0.2615	
St, Dev			0.1824	0.1517	0.15	0.0221	

N_A_: number of different alleles per locus, N_G_: number of genotypes detected in the 64 *Argyresthia conjugella* individuals from each locus, H_O_: observed heterozygosity, H_E_: expected heterozygosity, Nei: genetic diversity estimated (Nei 1975), F_IS_: inbreeding value, HWE: significance of departure from Hardy-Weinberg equilibrium, * < 0.05, ** < 0.01; ^a^ Based on assay of 64 *Argyresthia conjugella* individuals from each locus.

**Table 4:** Analysis of molecular variance (AMOVA) of *Argyresthia conjugella* among the three sampling regions using 259 alleles.

Source of Variation	d.f.	Sum of Squares	Variance Components	Percentage of Variation
Among regions	1	43.944	0.36434	2.17
Among populations with regions	4	48.2872	0.78612	2.24
Within regions	57	949.6497	15.47801	95.60
Total	62	997.937	16.26421	

**Table 5:** Pair-wise F_ST_ values among the three sampling locations of *Argyresthia conjugella* in the study.

Regions	East Norway	Sweden	West Norway
East Norway	0.00000		
Sweden	0.01577	0.00000	
West Norway	0.02522	0.01373	0.0000

## Supplementary Materials

The following are available online. Table S1: List of 64 *Argyresthia conjugella* individuals used in the study, Table S2: Allele frequencies for the 19 STR markers, Table S3: DNA sequence analysis, Figure S1: DeltaK for samples from 64 individuals indicating the number of clusters in the program Structure.
