# Supplementary material for: Monitoring of the Apple Fruit Moth: Detection of Genetic Variation and Structure Applying a Novel Multiplex Set of 19 STR Markers
Source: Molecules. 2018 Apr 8;23(4):850. doi: 10.3390/molecules23040850 (PMC6017289; doi:10.3390/molecules23040850)
Supplement: Supplementary file 1 [file molecules-23-00850-s001.pdf]

Supplementary data

Table S1. List of 64 *Argyresthia conjugella* individuals used in the study. F = female and M = Male.

Accession nr.	Country	County	Municipalat	Latitude	Longitude	Gender
15AC012	Norway	Akershus	Fagerstrand	59° 748'N	10° 587'E	F
15AC064	Norway	Akershus	Fagerstrand	59° 748'N	10° 587'E	M
15AC065	Norway	Akershus	Fagerstrand	59° 748'N	10° 587'E	M
15AC066	Norway	Akershus	Fagerstrand	59° 748'N	10° 587'E	M
15AC067	Norway	Akershus	Fagerstrand	59° 748'N	10° 587'E	F
15AC068	Norway	Akershus	Fagerstrand	59° 748'N	10° 587'E	F
15AC069	Norway	Akershus	Fagerstrand	59° 748'N	10° 587'E	F
15AC013	Norway	Aust-Agder	Longerak	58° 754'N	7° 846'E	M
15AC001	Norway	Hordaland	Åsen	60° 564'N	6° 930'E	F
15AC002	Norway	Hordaland	Torpe	60° 386'N	6° 221'E	M
15AC004	Norway	Hordaland	Djønno	60° 466'N	6° 750'E	M
15AC005	Norway	Hordaland	Alsåker	60° 389'N	6° 504'E	F
15AC006	Norway	Hordaland	Sekse	60° 246'N	6° 624'E	M
15AC054	Norway	Hordaland	Sekse	60° 246'N	6° 624'E	M
15AC055	Norway	Hordaland	Sekse	60° 246'N	6° 624'E	M
15AC056	Norway	Hordaland	Sekse	60° 246'N	6° 624'E	M
15AC057	Norway	Hordaland	Sekse	60° 246'N	6° 624'E	M
15AC058	Norway	Hordaland	Sekse	60° 246'N	6° 624'E	F
15AC059	Norway	Hordaland	Sekse	60° 246'N	6° 624'E	F
15AC061	Norway	Hordaland	Sekse	60° 246'N	6° 624'E	F
15AC062	Norway	Hordaland	Sekse	60° 246'N	6° 624'E	F
15AC063	Norway	Hordaland	Sekse	60° 246'N	6° 624'E	F
15AC007	Norway	Hordaland	Store Linga	60° 260'N	6.080'E	M
15AC011	Norway	Hordaland	Jaastad	60° 347'N	6.624'E	F
15AC008	Norway	Sogn og Fjordane	Leikanger	61° 188'N	6.803'E	M

15AC009	Norway	Sogn og Fjordane	Stårheim	61° 923'N	5.776'E	F
15AC010	Norway	Sogn og Fjordane	Sogndal	61° 235'N	7.116'E	M
15AC003	Norway	Sogn og Fjordane	Slinde	61° 162'N	6° 986'E	F
15AC014	Norway	Telemark	Haukvik	59° 381'N	9° 268'E	M
15AC021	Norway	Telemark	Haukvik	59° 381'N	9° 268'E	M
15AC022	Norway	Telemark	Haukvik	59° 381'N	9° 268'E	M
15AC023	Norway	Telemark	Haukvik	59° 381'N	9° 268'E	M
15AC024	Norway	Telemark	Haukvik	59° 381'N	9° 268'E	M
15AC025	Norway	Telemark	Haukvik	59° 381'N	9° 268'E	M
15AC026	Norway	Telemark	Haukvik	59° 381'N	9° 268'E	F
15AC027	Norway	Telemark	Haukvik	59° 381'N	9° 268'E	F
15AC028	Norway	Telemark	Haukvik	59° 381'N	9° 268'E	F
15AC030	Norway	Telemark	Haukvik	59° 381'N	9° 268'E	F
15AC031	Norway	Telemark	Haukvik	59° 381'N	9° 268'E	M
15AC033	Norway	Telemark	Haukvik	59° 381'N	9° 268'E	M
15AC034	Norway	Telemark	Haukvik	59° 381'N	9° 268'E	M
15AC035	Norway	Telemark	Haukvik	59° 381'N	9° 268'E	M
15AC036	Norway	Telemark	Haukvik	59° 381'N	9° 268'E	F
15AC037	Norway	Telemark	Haukvik	59° 381'N	9° 268'E	F
15AC038	Norway	Telemark	Haukvik	59° 381'N	9° 268'E	F
15AC039	Norway	Telemark	Haukvik	59° 381'N	9° 268'E	F
15AC040	Norway	Telemark	Haukvik	59° 381'N	9° 268'E	M
15AC041	Norway	Telemark	Haukvik	59° 381'N	9° 268'E	F
15AC042	Norway	Telemark	Haukvik	59° 381'N	9° 268'E	M
15AC043	Norway	Telemark	Haukvik	59° 381'N	9° 268'E	F
15AC044	Norway	Telemark	Haukvik	59° 381'N	9° 268'E	F
15AC045	Norway	Telemark	Haukvik	59° 381'N	9° 268'E	F
15AC046	Norway	Telemark	Haukvik	59° 381'N	9° 268'E	M
15AC047	Norway	Telemark	Haukvik	59° 381'N	9° 268'E	M

15AC048	Norway	Telemark	Haukvik	59° 381'N	9° 268'E	M
15AC049	Norway	Telemark	Haukvik	59° 381'N	9° 268'E	M
15AC050	Norway	Telemark	Haukvik	59° 381'N	9° 268'E	F
15AC051	Norway	Telemark	Haukvik	59° 381'N	9° 268'E	F
15AC052	Norway	Telemark	Haukvik	59° 381'N	9° 268'E	M
15AC015	Sweden	Skåne	Stenshovud (Simrishamn)	55° 656'N	14° 267'E	M
15AC016	Sweden	Skåne	Källagården (Brösarp)	55° 721'N	14° 103'E	F
15AC018	Sweden	Skåne	Svinaberga (Kivik)	55° 673'N	14° 240'E	M
15AC019	Sweden	Åland	Saltvik (Åland)	60° 274'N	20° 064'E	F
15AC020	Sweden	Åland	Finström (Åland)	60° 256'N	19° 902'E	M

Table S 2. Allele frequencies for the 19 STR markers. Fequencies were based on 64 *Argyesthia conjugella* individuals collected in Norway and Sweden in 2015.

Alleles frequency	Argcon891	Argcon3606	Argcon3484	Argcon886	Argcon3887	Argcon20889	Argcon1863	Argcon384	Argcon5649	Argcon4899	Argcon14321	Argcon17958	Argcon8461	Argcon1615	Argcon8345	Argcon1452	Argcon373	Argcon1132	Argcon3813
**Allele1**	0,0079	0,5385	0,0547	0,0391	0,0089	0,0391	0,0182	0,0081	0,1875	0,0089	0,0156	0,0077	0,0093	0,1053	0,0081	0,0417	0,0081	0,0081	0,1532
**Allele2**	0,0238	0,1000	0,0547	0,0391	0,0089	0,5625	0,0364	0,0242	0,0547	0,1964	0,1250	0,4538	0,1944	0,0351	0,0323	0,0972	0,0403	0,0968	0,1048
**Allele3**	0,0079	0,0077	0,3906	0,0078	0,0714	0,1250	0,0636	0,4597	0,0859	0,2321	0,1094	0,0615	0,0093	0,0614	0,2177	0,0556	0,1129	0,5242	0,6452
**Allele4**	0,1032	0,3000	0,0156	0,6875	0,0089	0,1719	0,0182	0,1371	0,3438	0,2411	0,3906	0,0077	0,0093	0,1316	0,2339	0,1250	0,1452	0,0323	0,0484
**Allele5**	0,6032	0,0231	0,1562	0,2188	0,0089	0,0547	0,0455	0,0726	0,1094	0,0625	0,0625	0,0692	0,0185	0,2193	0,1774	0,0556	0,0887	0,1532	0,0161
**Allele6**	0,0238	0,0231	0,0234	0,0078	0,1071	0,0234	0,7091	0,0161	0,1328	0,0536	0,2734	0,1154	0,0278	0,0088	0,0161	0,1667	0,0726	0,1210	0,0081
**Allele7**	0,1111	0,0077	0,1875		0,0625	0,0156	0,1091	0,0161	0,0234	0,0179	0,0156	0,0462	0,0278	0,1140	0,0081	0,0694	0,0726	0,0081	0,0161
**Allele8**	0,1032		0,0312		0,0446	0,0078		0,0726	0,0391	0,0179	0,0078	0,0615	0,1019	0,0175	0,0726	0,0417	0,0242	0,0081	0,0081
**Allele9**	0,0079		0,0547		0,0179			0,0565	0,0078	0,0268		0,0538	0,0278	0,0526	0,0565	0,0139	0,0565	0,0081	
**Allele10**	0,0079		0,0234		0,0446			0,0242	0,0078	0,0179		0,0077	0,1111	0,0088	0,0242	0,0972	0,0726	0,0081	
**Allele11**			0,0078		0,0268			0,0161	0,0078	0,0089		0,0077	0,0185	0,1053	0,0081	0,0278	0,0484	0,0323	
**Allele12**					0,0625			0,0323		0,0179		0,0462	0,1389	0,1053	0,0161	0,0972	0,0403		
**Allele13**					0,0268			0,0323		0,0179		0,0154	0,0093	0,0263	0,0565	0,0417	0,0403		
**Allele14**					0,0446			0,0081		0,0089		0,0231	0,0185	0,0088	0,0242	0,0278	0,0403		
**Allele15**					0,1161			0,0161		0,0268		0,0077	0,0185		0,0161	0,0139	0,0242		
**Allele16**					0,0625			0,0081		0,0089		0,0077	0,0093		0,0081	0,0278	0,0081		
**Allele17**					0,0357					0,0089		0,0077	0,0185		0,0081		0,0161		
**Allele18**					0,0446					0,0089			0,0185		0,0161		0,0323		
**Allele19**					0,0536					0,0089			0,1111				0,0323		
**Allele20**					0,0357					0,0089			0,0185				0,0161		
**Allele21**					0,0446								0,0185				0,0081		
**Allele22**					0,0179								0,0093						
**Allele23**					0,0089								0,0093						
**Allele24**					0,0089								0,0463						
**Allele25**					0,0089														
**Allele26**					0,0179														

Table S 3. DNA sequence analysis.

Locus	SSR primer sequences 5' – 3'	Sequence and STR Markers for *Argyresthia conjugella* based on genomic DNA sequence analyzed on an Illumina MiSeq platform. Primer positions(blue) STR repeat motif (red)
Argcon_373	F:AGTACCTCGTCGATACGCAC<br>R:AGGGGTGTCAGGATGTGATG	ATGACCTTTCGACCGCCTGTTTTCTAAAAATCCAAGAAATAT TTTCAAATTATACCAAGTTTGGACATAAATTGGTTCACAGTT GGTTGGCGAACAGGGGTCGCGAGGTCTACTGCGCACAGG GCCACTAGTCTTTACAATTCACTTTTATTATGAAAATGTTCAT TAAACCCGCAGGGCCAGTGAACAAGAGCATCCCATCCATC GTGCGTATACGCACATCCTCCGAGTCGCAGAACCTTGTCTR CGCCGAGCAAGAGACAGACGAGCTGATCGCCATGCTCCGG GAGACTGAGAGCTTGGACGAGCAGGGAGACATACTGCAGT ACCTCGTCGATACGCACGGCCTGGAGTTCAATTCTGGTGAG ATTGTTTGTTTGTTTGTTTGTTTGTTTGTTTGTTTGTTTGTTTG TTTGTTTGTTTGTTTGTTTGTTTGTTTGTTTGTTTGTTTGTTTG TTTTATAAGGTGTTTATCATCATCATCACATCCTGACACCCC TCAGCAGGGGTATAGGGCTCTCAGTACAGATTTCCATTTGT TCCGATCTTGGGCCATCTGCTCYAGCTCCGCCCAATCTGAG YTCACAATGGCRGCCTCCTTCTCCGTTGAACGTCGCCACGT TGTTTTGGGGCGACCTTTCTTCCGTTTCCCTTCTGGACACC ATTTGAGGGCTACTTTTGATAGATGTTCATCGGGTCTCCTCA TTACGTGGCCAAGCCAACGCAACTTTCTGGTGATCACCTCC TCCTCCAACGGCTTCTGATTCGTAAGTCTCTACAATTCCACG TTCGTAATCGTTTTTTGGCCAGAAAATTCTCAGTATCTGTCT CAAGCATTTGTT
Argcon_384	F: CATGTCTCCTCTTTGCAGCG<br>R: GTAAGGGAGTGTCGTGTTGC	ACACATCTCGTCATATACGACTATGAAAAGCGACATGCCTC TATTCAAGGCACTTGCATGCTAATGACAGACTCTAGCGTTTA AAGACACAACAAGCATACACGAGAACAACATGCATCTCATA AGCACATAGTAAGAGAGCAAACTGTGCTCACTGCTCACTGA ACAATTCAAGCGGTTCGGGATCTATGTGCTAACAAATACATA TAGTTCCTGTTTTTGCGGAGTGATCTGGCTTTATATCAGTAG CCTGTTCATGTGGTACAGCTGTCCCGTGACGTCACATGTCT CCTCTTTGCAGCGCAGATCCCGGCTGCAGATAGCGGTGTTT CTCGCGATGTCTCTCCGATGCCGCCCGTCTCCCCAACAAAC AAACAAACAAACAACAAACAAACAAACAAACAAACAAACCGCAAC AAGCAACACGACACTCCCTTACTCACTGCAACATTCAAGAT GCCTCATCCGTTTCCTCCGCAACAGAGTTGCTTCTAGCAAG ATTGAAGATAGCCTCACCGCGTCTAGRTGTGAGTTAGTGTA CTCGACTCCGATCTTTTTATTATTACCTATTGATTTTTGTTTC GTTTCG
Argcon_886	F: ACCCGACCTGAACATATCCG<br>R: CCATCGTTGGCACTTACGAG	ATATTCGGTATTCGGCCGAATCGTAGAAAGTATTCGGCCGA ATACCGAATAGTTGCCGAATATTTGTTGCATCTCTATTATGC

		ACAGTCATTGTATTGTTTGTACACATGTACATATGTAACAAG<br>AATAGTAATTGAATTTTTCGCGGTCACCACCCGACCTGAAC<br>ATATCCGCAAAACATTCGCTTCATTTGACACGAGTGAGAAT<br>GTGAATGTCTATGCGAATGTTCAAAATGCTGCGATTGCGAA<br>TATCCGCGCCATCCCGTGTGCTCACTGTCTTTGTAAATAAA<br>CGTTTCTTTCTTTCTTTCTTTCTTTCTTTCCCTAGCCTCGTAA<br>GTGCCAACGATGGAGACAGTTACCCTATCTTCACAAACCGA<br>TAATGGACTGT GAAGTCT
Argcon_1132	F: TGTCTATGGAAGCCCCGATG<br>R: GTTCCAAGATTTGCCGCTCC	AGGGTTAGGTATAATTAGGTTAGGTACCTTGAGGATCTTCAT<br>AGAAGCCTTGTCTATGGAAGCCCCGATGGGGGTCAGGGCG<br>TACTCGGTCTTCCCGAAGAGAGGAGTCGCCGAGCTGACCG<br>CAGCTGCAAAAAATATTTATCAAAACTCGGTTCAGCCGTTTG<br>GTTCAACTACTAACAGAAAGAAAGAAAGAAAGAAAGAAAGA<br>AAAAGCATGATGTTCTCTTTTTAACACCCTTCTATTAAGTCG<br>TGTTGTTACTAAATAACACGGAGCGGCAAATCTTGGAACGG<br>TAATTTCACCCACTTCTAGTTCTTCTATCATTATGAAACTYGG<br>GGTGTGTAGGTATGAGTTCCGATGACAATACAATATTATGG<br>TACCATCGAGCTGGTCTGATGATCGAGCTCGAAGGTGGCA<br>GAGGGAACCCTGGGATGGTTATTTGTAAATCCGTCGAGTTT<br>GGGCTCATTCGATTCGTCTTGATAGGACCTCTTGATCTCGA<br>AAGGGTGTTTT
Argcon_1452	F: AATAACAGTGGCACACCACG<br>R: TATGCGGATTCCAAACGCAG	ATGGATTGTACCACGCCGCAGTCTGTGATGCTGGCGAGTT<br>GGCGCTGACTGAAAATCAGAGTCAGACGCCATTTATAATAA<br>CAGTGGCACACCACGACAGTCATTATTATTACCGAGTCAAA<br>TCGATGAAAATTTACATTTGTGTTTTTGGAAAATTGACAGTC<br>ATTTCTACTTTCTGTGTTTTGTTTGTTGTATTTGTTGCTATTG<br>TAATGTTAGTGTGTTCACTGTCCTTATAATAAAAATTTCTATC<br>TTTCTTTCTTTCTTTCTTTCTTTCTTTCTTTCTTTAAGTTTGCA<br>TCTAGTCTGCGTTTGGAATCCGCATACTTGTGTGTCCGCGT<br>CGTCGATTCGCGTTACATCGCCTTGCCCGCGACTTATGTAT<br>TTAGAATCGACGGCGCGGCACAGAATAACACTGTCGCGGC<br>AAGGCGAATCGACGTCGCGGAAACCAATATTATTATTCAGC<br>CCTTATAAAACTCAAATCTTCGTTGGCTTATGGGATGGAAAT<br>TATTACTCGTAAAATCGCTACGCTAAATCTCACAAATAATAC<br>ATTTGACTCCAGCTATCACCGGTAGCATGGAGTGTG
Argcon_1615	F: CCCCTTATTTGAGCAGTTGAGC<br>R: GCAACATTATTTCGTCCGCAG	ACGATATAGTCTTTACTGTGTTCTATTATTCTACACATCAACA<br>TAGTAACATGCTTTTGCATAATAATATGCCTTCTGCTCCACT<br>TTTACAGCGGGTATTATACTGGGAGTTATAACGGAAGGTGT<br>TTATAATTACAATTTGAATTACAGTTTCTATGTATAGATTTGG<br>CCACAGTCCGTATTTGTATTAATCAAATTCAAGAAACTCAAG<br>GAAGCATGTGACACAGAATGTACCAGGAAATACTAGGTACC<br>TTTTTGTAACACCCCTTATTTGAGCAGTTGAGCTTACCTAAT<br>AGTACATAGGTATAGAAATTTTACACCATAGAGCCTTATTTT

		GTTTGTTTGTTTGTTTGTTTGTTTGTTTGTTTGTTTGTTTATG GCTGAATTTTATATGTCTGCGGACGAAATAATGTTGCAAGTT CTTTTAAACTCCATTATAATATAATAACAAAGCT
Argcon_1863	F: CGCCCCGGATTCTCAACTAC<br>R: TCACCCCTCTCTGTATTCGTC	GATATAGGGAACTATCGCCCTACTAGTTTAGTTTCGCATATC TATACAAACTATTCACTCGTACCGACTTATCCTAATTGCCCT GAAACTGTATTTCAGTACAATTAGTTTCCCATCAACCCCCAG AACAGGCTGGTTTCCGCCCCGGATTCTCAACTACAGACCAA CTACACACATTTAACCGGATAATAGAGAAGTATACTGAGTTC AACAAGCCTCTCTTCTCTACTGAGGGTTTGTTGACTACTCAA AAGTTTTCGACAGTATCACTCACTCATCAATCTTTACCGCAC TTCACAATCAAAATATAGATCCCACATACATACATACATACA TACATACATTTATATAATGATATGAGAGAGACGAATACAGAG AGGGGTGAAACAGGAAGACCCYCTATCTCCATAACTATTTA CATGTAATGAAGAAGTTTTTAAAAACTTGGCGGTCCCGTGG CACAGCAGAGGAATAATTGTCGTYGGCGATAAAAGGTTGCA TAACTTCGCTTTGCCGATGACATTGTTCTATTCGCGCCATCC TCGTCAGAACTCAGAGAGTACATCTACTAAAACAGAGAGTA CATCTACTTGGGCCAGACAGTTTCCTTCGAGAAC
Argcon_2891	F: AGAACTGGGCCTCACGATAC<br>R: GTTATCGGCATTCCACAAGGG	ATAGTACTATCGCTATGTACTTGTCGTAAGTTAAACATAAAT TGTAATTCCTATGTCAAGAACTGGGCCTCACGATACAGGTC TAACCTAGTGTGAGGGCCGCMGGTAATCAATATTACTCCTT AGAAAATACGAACCTAATTAATAAGTGAATTTGTATWTTATT TTGCTAAATAAACGATTATCTATCTTATCTATCTATCTATCTA TCTATCTATCTTATTAGCTGGTATTTTAGTATAATTTCTAAGC CTCTGCTGCCGTTTGATCAAAGTTCAGATAACAACCCTTGT GGAATGCCGATAACTTCTCAAAGGATTCCATGAAACCTAATT ATTTAGTAGCATTAAATTGATAATACTCTAATCGTGTTTTAGA AACGAAATTAAACTAAGCCGAGCTTAGAACCCGTTGATCAA TTCTGTTGAGCCAATCGAAGTGTATGATCAAAGACTCGTGG TCTGGCGTGGGGGGTTATGAATTAACATTCACTAAAATACA
Argcon_3484	F: GGGCAGCTGTTTCCCAATTCC<br>R: CTCCTCGTGCATTTTTGGG	CAACTCCCACGAGAGAAGCAAGCATGAAACCCACCAGACC ACAAAACTCTTTTAAGCATTTTTTTATTTTACAAGTATATTTAT CTGCCGTCTAAACGCACTTCCCGGGCAGCTGTTTCCCAATT CGCAACTTCTAAACGCTGCCTTCGATATAAATTTTTCCTCCA AAAGAACCAAGTTTGTTAGTTGTTTGATAAAGTTCGGAATTA ACATCAAGTCGAAAAGTCTTGAGCGGTCAAGTGCGGAGAG ATGAACGGCTCCCGAAAATTTAACTCGGTTGTTTGTTTGTTT GTTTGTTTGTTTGTTTGTTTGCCGCGATAATACAGTAAAACT AACCCAAAAATGCACGAGGAGGCCTACACTCAGCAGTGGG CTCCCTGACTAGCTGACGATGAAGATGATAAACCCAAAAAT GGGCAAGCTGCGGAACTAATT

Argcon_3606	F: AGTGAACCTACTGAGCGTCC<br>R: CCCTTATGGGAAAAGGCGTG	GACTGAGTTGGGAAAACCTACTATGGATTTTAAAATCTGTCT<br>GACTTGGGATATGTCTGAGATGGGAACAACCCCTCTTTCGA<br>GTTTAGACYTCCCACGGCGGCGCTCAAGCGTCTTCTATCAA<br>GCTCAATCCCATTTACTTCTCCATTTGTCTCGCTGCTCGGTT<br>CGAGACGACCGTGTGAAAACACTCTTACAATAAAGTGAACC<br>TACTGAGCGTCCATTTTATCTTCTCTTGGTCCTAACCCATCA<br>AAAGAGCCCTTAGTGTTCAAACGGCCAACACTAGATTTGAA<br>TGTACAACATACATACATACATACATACATACATACATACATA<br>GCGTCACGCCTTTTCCCATAAGGGGTGGCAGAGGCACAAC<br>TGTGCCCACTTTCAGCCAGTGATTCAATAATTCATTTTGCGT<br>GACAGGGGGCTAGCCCATCGCTGCACAGCAGTTATGGAGT<br>AATGAAGGTTTCCTTACGATGTTTTCCATCRCCGTTAGAGTG<br>TCCGTACAACGTACTTAAGATAAATTACWTAWCTCATTGGT<br>ACATGGTCAGCACCGTGATCAAACCTGCATCTCCTGATTCA<br>GGCGTGAATCCCACTAGACCACGGATCTCTTTCGCGT
Argcon_3813	F: GCTGTCGTAAACCCTTCCAC<br>R: GGCCCCATTGGTTCCATAAC	AACATTACTTGGTAGTAGTAGCTGTCGTAAACCCTTCCACCA<br>AACCGCACTGAGCCAGCATGGTGGAGAAAGGCTCAGTCTT<br>ACTCGTATCCGGGTCAAGGCCTGTGCCCCTCGGTGGAAAT<br>ATTAATGTGCTACATTAACTTATGTGGCAAATAGGTGCTTCT<br>TCTTTCTTTCTTTCTTTCTTTCTTTCTTTCAGTTATGGAACCA<br>ATGGGGCCATTTGCTTTATACTTTACAAAAAGTGGTCAATGT<br>TATCCCAATGAACCGTGTGTAGAGGGAGGCACAATCGAACC<br>AATCACAGCGTCAATTTATTCAGCTGTAAAATCAGTGTTGTG<br>TTCCACAATATCATTTTCTGTTATTCGAAAACCAA
Argcon_3887	F: CATTGTTGACAGCTCGGCAC<br>R: GTGAGCCTTTCGGATTTGGG	ATCCACTATGTATCTGCCTCGGCCGCCCGATGTCGCTGTCC<br>GCAACATTTTACTAATACGATGTGCCGAGCCGGCAATATTG<br>CCATTTTTACCGGCAATATTGGCATTTTTTGTATGGGGCAAT<br>ATTGCCGGCAATGTTGCAAAACATGGCARTTTTGCCGCATT<br>GTTGACAGCTCGGCACATCTATATGTATACTACACATCTTAT<br>TTTTATTTAGGTAAGTACACTAAAAAATAAAAAATAAAAAATA<br>TATCAGGTATCAATCATCTATAAATCATTCAAGAAAGAAAGR<br>AAGAAAGAAAGAAAGAAAGAAAGAAAGAAAGAAAGAAAGA<br>AACGTTTATTTCAGGGCGACTTACCCAAATCCGAAAGGCTC<br>ACTTTCTCGAGGAAGGGATCTGCTACCGGGAGCTGCGGGA<br>GGAAA
Argcon_4899	F: TCATGGTTTGGCATGTCGAG<br>R: AGTTCAAATCCGTCCTAAAAGC	TCCACTTTTGTCAGAAACCGCCATTGCTTTTATCCGTTGATC<br>AAATAGATTCATGGTTTGGCATGTCGAGCTAAATATAGCCAA<br>AACAAAAACATACCAACTCGTATTTTTCCATATGCAGAGGCA<br>CGGATAAAATAAATTATAGATATCATTTACCTACAGTTGGTA<br>CGGTGCAATGCTTAAATAAATGTTCTTTCTTTCTTTCTTTCTT<br>TCTTTCTTTCTTTCTTACAAATGTTTTTTTCAAATTTCTGGTAA<br>AAAGATAATTGTCAAGAGCTTTTAGGACGGATTTGAACTTGT<br>AAACCATATCCATCGAGACCATATCATACCTCATCATCTCTT

		CAAATACCGTAACATATTTAATAAAAGTTTTGTGCTTAGTAAA<br>CT TGGTTAGCAAGACCTATATTATTATCTCGCTCGCTTGC
Argcon_5649	F: AGCCCTACGACTCCATCAAC<br>R: GCTAAACTATCCGTCGGCAC	ATGGCGGCCGGCCTCTACCCGCCCGGCTACCCGCGCCAG<br>CCCTACGACTCCATCAACAGACCCTCCTGGCTCTAAACAAA<br>CAAACAAACAAACAAACAAACAAGCAAGCAAGCAAACTGCT<br>AAATTACTTTACTACTACAACAACAGTGCCGACGGATAGTTT<br>AGCTCTACCGCCCCTCTGACCACGCGCCTTCAATGTCTATA<br>ATAGTAAATCAGATTATTCTACATAGATRATAATACCATGGT<br>ATTAATTTATTATATTGTTTGTAGATTTAAAATAGCCCTTTGT<br>GAATTAATA
Argcon_8345	F: GCTCAAACGGTTGTCCCTAC<br>R: GTATGCTACGGTTACAGGGC	CTCAATATGAATCCGATTTGATCAGAGCAACTTAATTGATCA<br>CTTCTTCATACAAATCAGCTTATCATACAGTCTTATAAGTATA<br>AATATTGGATTTTAGACTGAATGGGTCAGCTGATTTGTATGG<br>AGAAGTGAACAATTAAGTTGCTCTGATCAAATAGGTTTCACG<br>GTGAAAGTTGCTCAAACGGTTGTCCCTACAACCCCTAGATA<br>CCTAGGGGTACGTAGTAAACAATTTCTTTTTTATATTGTTTCA<br>CATAGGTGTTTTTTTCGATTTTGTTGTATGTAGTAATAAACG<br>CAATTTCTTTCTTTCTTTCTTTCTTTCTTTCTTTCTTTCTTTAGGGGT<br>TTTAGTTTCACCTTGTATTTTTCTGTAGAATCGGCCCTGTAA<br>CCGTAGCATACTGATGCAGTCCAATACAATCTCGACCACCA<br>CTATATTTTTTAACCCCCGCCCGACGAAGAAATAAGGGGTG<br>TTATAAGTTTAAAGTGTGAGTCACTCTGTCTCAGCACGAAGA<br>GAGTAAACGGGTGAACCGATTTGAATGTTATTTTTTTATTCG<br>AAAATAGATTTCTTGGAGCTGGTTGCYATGGTACCATCGAG<br>CTGGTCTAATGATGAAGTTCAAAGGCGGGAGCGGGAACCC<br>TATTAGTATACCTGTCGAATTTGGGCTCATTAGGTTCGTTGT<br>GACAAGACCTTTA
Argcon_8461	F: ACTTTACTGGCCTAGGTGCG<br>R: CCAGGTGAAACATCGTGAGG	GCTTTTTCTGTCCTCCGGCCAGAAAGCGTCACTTTTCTGGC<br>CGCTGCGCTAGATAAAGTTGCCGTTTTTTGGCTCCGTCGAA<br>CAGAAATTTGTATATGACCTCGGCCAGTAAATAAGAAAGCC<br>TCAGATCACATGTTCGTCGACCTCGGCTTCGCCGCCAATAA<br>TTACATGTGATCTGAGACTTTTCTTACTTTACTGGCCTAGGT<br>GCGTAATGTACTATTTATGTTTTAGTTTGCGGAAAATGAACG<br>TTTCTTCTTTCTTTCTTTCTTTCTTTCTTTATTTCTTTCTTTCTTTCTT<br>TCTTTCTTTCTTTCTTTCTTTCTTTCTTTCTTTCTTTCTTTCTTT<br>CACTTTCAGTCAGCCATCGCCGCAGCTATGGAGTAATGAAG<br>GTTTCCTCACGATGTTTCACCTGGTTATACGACTAACGATTC<br>GCCGAGACTCGAAGGGCAAGAGCAAGGCTCGGCCATCGAT<br>CACGGCATCGGGGTCACGAGTTTTTTCGAGTATTCGTGAAC<br>GAGCTAAAGTAGGAGAGGCGGCGGGAAATGCATGCGTGTG<br>TAAGCTACTATAAAAATT
Argcon_14321	F: TGTTTTGTTCAATCTGTATTACTTGTC<br>R: ACAGGGGGACAATCCAATCTAC	ATACTTATGTTTTGTTCAATCTGTATTACTTGTCTATTTGTGT<br>GTATTCTTACAGTTAATAAACGGATTATCTATCTATCTATCTA

		TCTATCTATCTATCTAATATGAAAATGCGGGTATGGGTAATA TTGTATAGTGTTCGTTACTACTCCGTCTAATCTTCCTTAAAC ATTGGGTTTTTGAATGTTGTTCTAGCTTCTTTGTAGATTGGA TTGTCCCCCTGTAGAGAAGAAAAATACAAATGTAAGTAAGTA AGTAAGTAAGTTTATTTCAGTAAGCACAATAATATTACAATTT GTGGGTAGTGTCCCATACTAGAAAAATC
Argcon_17958	F: GCTCAGTGTATCAGGTACGAG<br>R: CGCTGTTCTACATGGAGCTG	GGACACAACGTGGTGCGTCCCATTCCGTATGGCAAAAAGT GTGATGTAGGTATCTTTGGTGTTGTTTCTGTGTAAGATAAGA AGCTCAGTGTATCAGGTACGAGTAGGTTTGTTTGTTTGTTTG TTTGTTTGTTTGTTTGTTTGAGTAGTGATAAGTGTTGCTTGCT AACCCTTGAGCGCTGGACAGATGCGCTTCCAGAGCGTGAG GCAGCCGCAGCGGTGGAACTGGCCCAGCGCCAGCTCCAT GTAGAACAGCGGCAGCCCGCCGAACAGCAGCATGATGCAG TACGGGATCAGGAACGCGCCTGCCATCCACCCACCACCAC ATTACCGGGACACGGAAAACACAACACAACACACAGACACG CGGTGATGCTACATGCACAAAAGATTCGGTGGAGAAAACTT ACTTACAATATTAATATAAGTGTA
Argcon_20889	F: TGTGTCTAGTTTCTTGTATTTGTTGC<br>R: TAGTGTGGGCTAAGGGATGC	TGTGTCTAGTTTCTTGTATTTGTTGCTTTTGTAATGTTAGGTT TACTGTCCTTTATAATAAATGTTTCTTTCTTTCTTTCTTTCTTT CTTTCTTTCTTTCTTTCTTTCTTTCTTGCATCCCTTAGCCCAC ACTATGAAGATGATTTGGTCAATAAAAAAATTGAACTCTGAA CTCTTAATTCTGCCTTAGACCCATTCAATAACCGATAAAACA TTACACTATAGCAAACTTTCCTTTCCTCGGGGAACACCCCCT CTACTCTCCCCATTTAGATATTATTTCGCAATATCGTATGTA GAGTAGTCTTTAGTTTTATAGTATCGGAGTACAAGTGAAGCC ATCTTCAAGGTTTTGACGTCTACTCCCTCCGATACTCCTAGC GATATTATGTCTAAAAATGGGATGCAGGGCTATCTAAAGTAT ATGTGAGGTAGTAAGTCGTAGTAAA

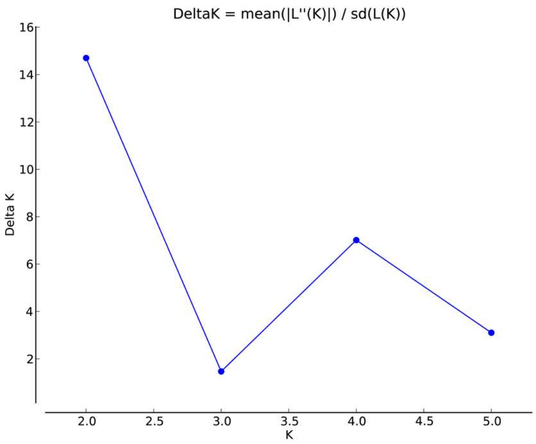

Figure S1. DeltaK for samples from 64 individuals indicating the number of clusters in the program Structure. DeltaK= mean (L′′(K)I)/s(L(K)).
